# Determination of the Geographical Origin of Coffee Beans Using Terahertz Spectroscopy Combined With Machine Learning Methods

**DOI:** 10.3389/fnut.2021.680627

**Published:** 2021-06-17

**Authors:** Si Yang, Chenxi Li, Yang Mei, Wen Liu, Rong Liu, Wenliang Chen, Donghai Han, Kexin Xu

**Affiliations:** ^1^State Key Laboratory of Precision Measuring Technology and Instruments, Tianjin University, Tianjin, China; ^2^School of Precision Instruments and Optoelectronics Engineering, Tianjin University, Tianjin, China; ^3^School of Chemical Engineering, Xiangtan University, Xiangtan, China; ^4^College of Food Science and Nutritional Engineering, China Agricultural University, Beijing, China

**Keywords:** THz spectroscopy, machine learning, classification, geographical origin, coffee beans

## Abstract

Different geographical origins can lead to great variance in coffee quality, taste, and commercial value. Hence, controlling the authenticity of the origin of coffee beans is of great importance for producers and consumers worldwide. In this study, terahertz (THz) spectroscopy, combined with machine learning methods, was investigated as a fast and non-destructive method to classify the geographic origin of coffee beans, comparing it with the popular machine learning methods, including convolutional neural network (CNN), linear discriminant analysis (LDA), and support vector machine (SVM) to obtain the best model. The curse of dimensionality will cause some classification methods which are struggling to train effective models. Thus, principal component analysis (PCA) and genetic algorithm (GA) were applied for LDA and SVM to create a smaller set of features. The first nine principal components (PCs) with an accumulative contribution rate of 99.9% extracted by PCA and 21 variables selected by GA were the inputs of LDA and SVM models. The results demonstrate that the excellent classification (accuracy was 90% in a prediction set) could be achieved using a CNN method. The results also indicate variable selecting as an important step to create an accurate and robust discrimination model. The performances of LDA and SVM algorithms could be improved with spectral features extracted by PCA and GA. The GA-SVM has achieved 75% accuracy in a prediction set, while the SVM and PCA-SVM have achieved 50 and 65% accuracy, respectively. These results demonstrate that THz spectroscopy, together with machine learning methods, is an effective and satisfactory approach for classifying geographical origins of coffee beans, suggesting the techniques to tap the potential application of deep learning in the authenticity of agricultural products while expanding the application of THz spectroscopy.

## Introduction

Coffee, as one of the most popular beverages in the world, is widely appreciated by consumers for its unique aroma, flavor, and refreshing effect (1–3). The sensory properties of coffee are profoundly affected by the composition of coffee beans, which are mainly affected by climate characteristics associated with different latitudes and altitudes. Central and South Africa offer optimal climate conditions for coffee plants. However, a great variance in coffee quality, taste, and commercial value is found with different geographical origins (4–7). Inevitably, this variability aspect might also increase the risk of fraud, such as mislabeling of the product to conceal the true geographical origin of the coffee beans (8). Hence, the development of analytical methods that could efficiently evaluate the geographical origin of coffee beans is highly encouraged by coffee producers and consumers.

Several analytical techniques, such as chromatography (9–11), electronic nose, and nuclear magnetic resonance, Flambeau et al. (12) have been applied to discriminate geographical origins of coffee beans by measuring physicochemical parameters, including caffeine, amino acids, chlorogenic acids, saccharides, and metal content (13–15). However, these methods are time-consuming, costly, and unsuitable for online applications. Due to the advantages of nondestructive and rapid, spectroscopy methods have been increasingly developed as a powerful analytical tool (16–20). For what it concerns the coffee production and consumption, Raman spectroscopy has been applied to discriminant Arabica and Robusta coffee beans (21, 22). For near-infrared spectroscopy, many applications for coffee beans have also been reported, such as discrimination of varieties (4, 23), prediction of roasting degree (24), and evaluation of coffee beans quality (18, 25). However, the superposition of different overtone and combination bands in the near-infrared spectroscopy region causes very low structural selectivity for NIR spectroscopy (26).

The frequency range of terahertz (THz) radiation is within 0.1–10 THz (27–29), where many fundamentals can usually be observed in isolated positions. Many organic molecules have strong absorption in the THz region due to the rotation and vibration transition of the dipole (30, 31). Meanwhile, the THz wave has relatively low-photon energy (4 meV for 1 THz) and strong penetration; which will not cause damage to biomolecules (32). In addition, compared with commonly used near-IR spectroscopy, THz wave possesses a longer wavelength and, therefore, cannot be easily influenced by scattering (33). Compared with Raman spectroscopy, THz wave is not easily affected by fluorescent substances in food (34). Thus, THz fingerprint spectroscopy becomes one of the most promising techniques for substance detection (29, 35). It is widely used in food quality and safety control, such as identification of floral resources of honey (36), discrimination of extra-virgin olive oil from different origins (37), detection of melamine in foodstuffs (38), and classification of transgenic food (39–41). However, promptly distinguishing the geographical origins of coffee beans in an effective manner using the THz spectrum combined with traditional modeling methods is still a challenge.

Machine learning, which is widely used in spectroscopy analysis, could also extend to THz spectroscopy data processing. Linear discriminant analysis (LDA) and support vector machine (SVM) have been proved as effective supervised classification methods in THz spectroscopy applications (37, 42). Owing to the multicollinearity and the interference of uncorrelated variables, most machine learning methods are based on spectral features rather than whole spectral data (43, 44). Recently, dramatic improvements in machine learning have mainly originated from deep convolution neural networks (CNN). Taking the advantages of effective structure and a convolution core in various scales, CNN can retain information of spectral features. Thus, the weak features can be enhanced even with a low signal–to-noise ratio (45, 46). However, CNN could automatically extract complex spectral features, making the classification model more accurate and robust.

In this study, we develop several methods that are based on THz spectroscopy, combined with machine learning, to classifying the geographical origins of coffee beans. In detail, CNN was investigated to simplify the feature extraction process while ensuring predictive precision and accuracy. Moreover, LDA and SVM were also applied to develop a series of classifiers with the spectral features selected by principal component analysis (PCA) and genetic algorithm (GA). The results provide a new idea and attempt for the application of THz spectroscopy and a machine learning method in food and agricultural applications.

## Methods and Materials

### Coffee Samples

Ninety-six samples of Arabica coffee beans, representatives of three different geographical origins, were analyzed in the present study. Out of these, 30 Kenya AA samples came from Muchagara Estate in southern Kenya, 30 Kilimanjaro samples came from Edelweiss Estate in Tanzania, and 36 samples came from Baoshan, Yunnan. The producing countries and the species were chosen according to their relevance to the Chinese coffee market. Africa is the region with the largest number of coffee-producing countries. High-quality coffee beans in Africa usually come from Ethiopia, Kenya, Tanzania, and so on. Kenya and Tanzania were among the top-20 producing countries. According to International Coffee Organization (ICO) (47), both Kenya and Tanzania contributed about 0.6% of the total world coffee production. The current production of coffee in Kenya and Tanzania is estimated to be 45,355 and 49,484 tons, respectively. Therefore, Arabica coffee beans from Kenya and Tanzania in Africa were selected for the research.

The roasting degree may cause chemical changes of coffee beans and influence the classification of geographical origin. To evaluate the classification model, samples from Yunnan were further prepared with three different roasting degrees (48), including light roasting (LR), medium roasting (MR), and deep roasting (DR). The roasting temperature is 200°C, and the roasting times are 8, 10, and 14 min, respectively. Meanwhile, Kenya AA and Kilimanjaro coffee beans were both prepared with MR degree. Because Kenya AA and Kilimanjaro coffee beans are imported from Africa, they have higher prices, and their taste is best under moderately roasted conditions. In terms of consumer habits, Kenya AA and Kilimanjaro coffee beans are both popular in the market for moderate roasting, whereas Yunnan coffee beans are sold in all three roasting methods. Meanwhile, Yunnan-Arabica is a domestic coffee bean, which is cheaper and easier to obtain. It is easy for unscrupulous merchants to use different roasting degrees of Yunnan-Arabica to pretend to be imported coffee beans to deceive consumers.

**Figure 1:**
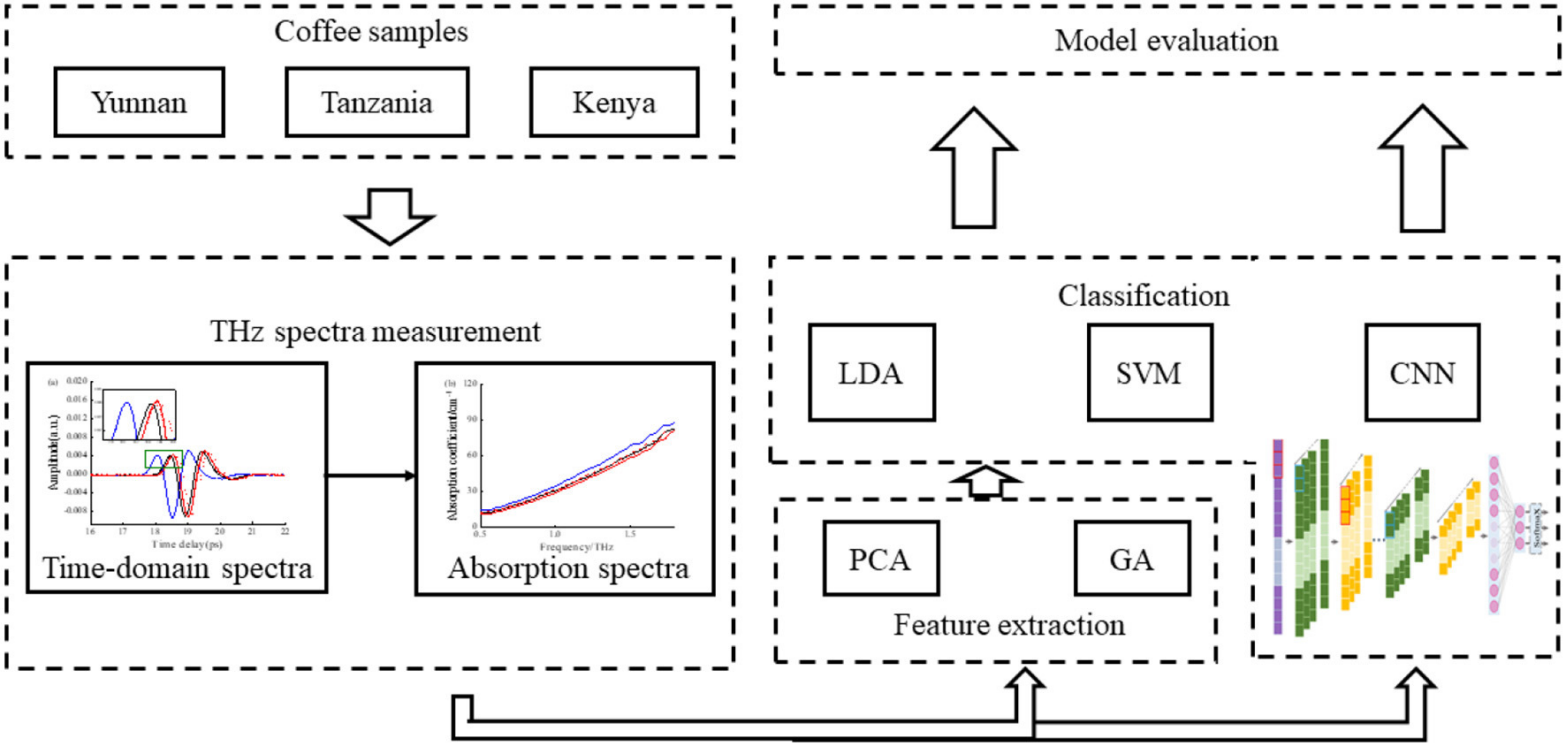
Main steps of classifying geographical origin of coffee beans.

Each coffee bean sample was first pulverized into flour by a pulverizer, which was then grounded to fine powder with agate mortar, and at last, pressed to a small tablet with a flat surface to eliminate the influence of multiple scattering. The tablet sample was finally made from 0.2 g of coffee bean flour; the conditions for pressing were 15 tons of pressure for 15 min. The schematic of the main procedures of the classification model is shown in Figure 1.

### THz Spectroscopy Measurement

The coffee bean samples were analyzed using a time-domain THz spectroscopy system (TAS7500SU, AdvanTest Crop., Japan) with a resolution of 0.0076 THz. Limited by a device, only the THz absorption spectral data within the frequency range of 0.5–1.9 THz were reliable. During the measurement, the optical cavity was filled with dry air to eliminate the interference of water vapor. Each spectrum was valued as the average of three measurements to improve the signal-to-noise ratio. The THz time-domain signal can be written as

(1) $$\begin{array}{r} S\left(t\right)=A\left(\omega \right)e^{j\left(\omega t+\varphi \;\left(\omega \right)\right)} \end{array}$$

where ω represents the frequency of the THz wave, *A*(ω) and φ(ω) are the amplitude and phase of THz-TDW, respectively. According to the optical parameter extraction model (49), the refractive index *n*(ω) and absorption coefficient α(ω)of a sample could be calculated as follows:

(2) $$\begin{array}{r} n\left(\omega \right)=\frac{\varphi \left(\omega \right)c}{\omega d}+1 \end{array}$$

(3) $$\begin{array}{r} \alpha \left(\omega \right)=\frac{2}{d}\ln\;\left\{\frac{4n\left(\omega \right)}{\rho \left(\omega \right){\left[n\left(\omega \right)+1\right]}^{2}}\right\} \end{array}$$

where *d* is the thickness of the sample slices, φ(ω) and ρ(ω) are the phase difference and the amplitude ratio between the sample signal and the reference signal, respectively.

### Feature Extraction

Some classification methods struggle to train effective models when the number of spectral features is very large, which is called the “curse of dimensionality” (50). This is especially relevant to algorithms that rely on distance calculations, such as LDA and SVM. Feature extraction is the critical step to avoid the curse of dimensionality by creating a smaller set of features that still capture most of the useful information.

Besides using the entire spectra (including 185 points), two feature extraction methods were evaluated in this study. First, PCA projects the original variables to a new coordinate to obtain a set of values of linearly uncorrelated variables called “principal components” (PCs) and thus eliminates the overlapping parts of coexisting information (51). Meanwhile, nine PCs with an accumulative contribution rate of 99.9% also provide information about the characteristic peaks. In the second feature extraction technique, the GA replaces the parameter space of the problem with the coding space (52). We implemented an adaptive GA that can automatically adapt the parameters of the crossover and mutation rate. The selected variables almost identify spectroscopy-relevant regions clearly after the evolving process. Since the GA is a mainly stochastic algorithm, each group of data runs at least five times. After the GA process, 21 variables were selected as the most streamlined and important variables.

### Classification Model

In this study, three different supervised machine learning algorithms, including CNN, LDA, and SVM, were investigated to classify different geographical origins of coffee beans.

**Figure 2:**
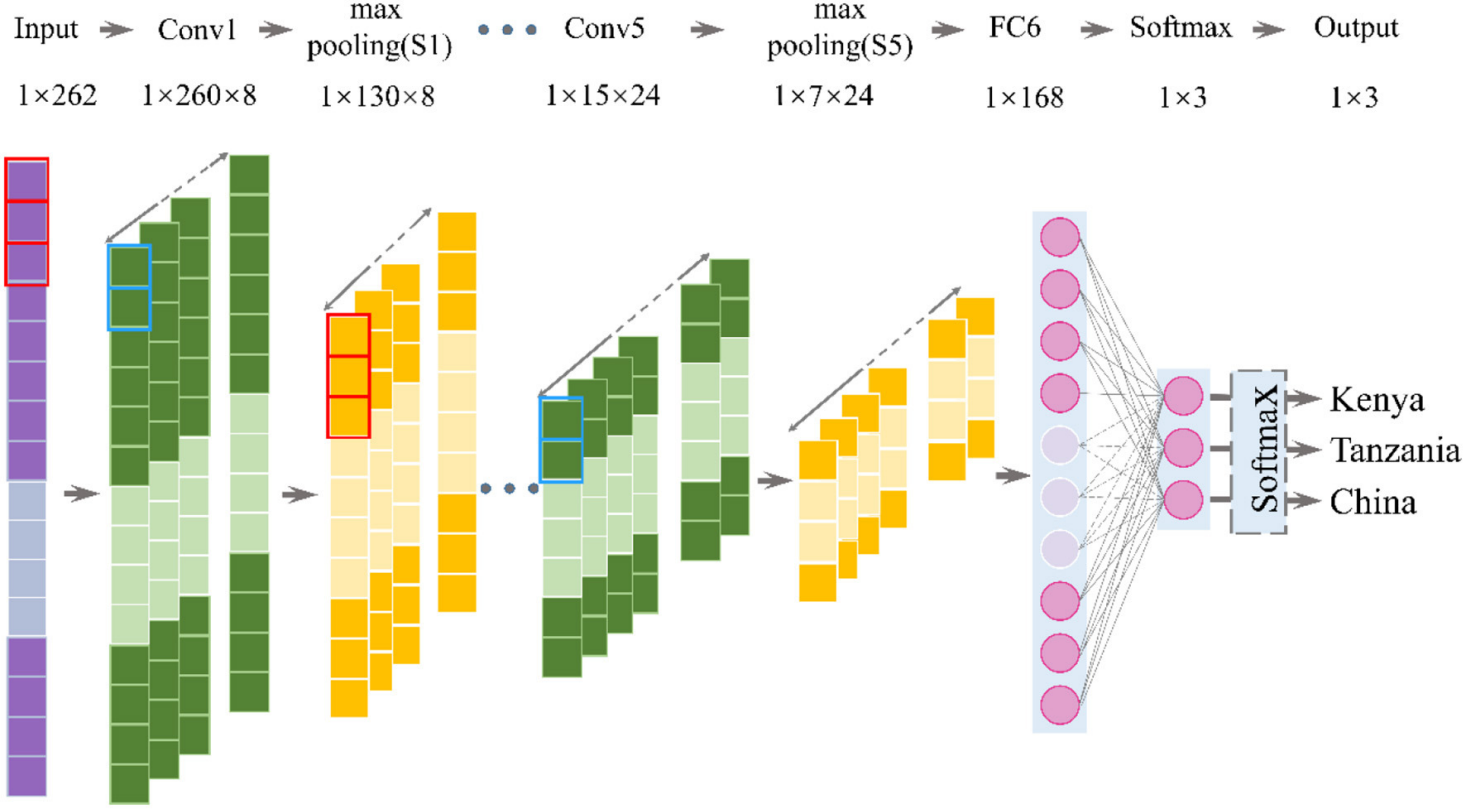
The hierarchical construction of the convolutional neural network (CNN)-based classifier.

Convolutional neural network is a special depth feed-forward neural network, which could work without any prior knowledge or human efforts in preprocessing raw data (53). The CNN algorithm essentially achieves the mapping of input to output by extracting features and reducing dimensions of the data (54). Figure 2 is the hierarchical construction of the CNN-based classifier, which consists of an input layer, a hidden layer, a full-connection layer, and an output layer (55). The parameters of each layer are shown in Table 1. The input layer size is 1 × 262. Based on the initial size of the THz spectrum, in this study, we choose a smaller convolution kernel size and a relatively deep network. In the convolutional layer, the kernel size is set as 3 × 1, and the convolution kernel of the set size is sampled according to the stride. The convolution operation is performed by multiplying the kernel by each point of the input data (56). The bottom convolutional layer can capture different low-level features, and the higher convolutional layer can capture more abstracted and discriminative features (57). The max-pooling layer was connected behind the convolution layer and was used to extract the invariant features, compress the feature, reduce computational complexity, and prevent overfitting, therefore increasing the overall performance and accuracy of the network (58). The kernel size of the max-pooling layer is set as 2 × 1. Selection on the number of feature maps, when the size of the output feature is halved, the number of the output channels should be doubled to ensure that the information contained in adjacent convolutional layers does not differ excessively. Through five convolutions and pooling operations, the extracted feature can be regarded as the abstracted and discriminative high-level feature. The increase in the number of convolutional layers will not improve the accuracy of the model, and the corresponding calculation amount and the time consumption will increase. Meanwhile, too few convolutional layers will result in imperfect feature extraction (59). All features will be reshaped into one-dimensional vectors and transmitted to the fully connected layer. In the last layer, the Softmax function was used to get the probabilities of each class of coffee bean. The deep network will improve non-linear fitting capabilities due to the nesting of multiple activation functions, making the network represent a wider range of functions. Relu is selected as the non-linear activation function; its unsaturation can improve the computing speed and better converge the network. Considering the problem of overfitting, we introduce the dropout method in the network, which could randomly discard some neurons in the full connectivity layer. The learning rate is set to 0.01. The cross-entropy loss was adopted as the loss function. By sharing weights and sparse connections, the CNN can greatly reduce the parameter size and the amount of training data (60, 61).

**Table 1:** Parameters of each layer in 1D-convolutional neural network (CNN).

**Layer**	**Type**	**Feature map**	**Kernel size**	**Stride**	**Dropout**	**Size**	**Activation**
In	Input	1	…	…	…	1 × 262	…
Conv1	Convolution	8	3 × 1	1	1	1 × 260	Relu
S1	Max pooling	8	2 × 1	1	…	1 × 130	…
Conv2	Convolution	12	3 × 1	1	1	1 × 128	Relu
S2	Max pooling	12	2 × 1	1	…	1 × 64	…
Conv3	Convolution	16	3 × 1	1	1	1 × 62	Relu
S3	Max pooling	16	2 × 1	1	…	1 × 31	…
Conv4	Convolution	20	3 × 1	1	1	1 × 29	Relu
S4	Max pooling	20	2 × 1	1	…	1 × 15	…
Conv5	Convolution	24	3 × 1	1	0.8	1 × 13	Relu
S5	Max pooling	24	2 × 1	1	…	1 × 7	…
FC6	Fully connected	…	…	…	1	1 × 168	Relu
Out	Fully connected	…	…	…	1	1 × 3	Softmax

Linear discriminant analysis achieves classification by searching for directions (canonical variables) that maximize the ratio between interclass and intraclass variances (62). SVM constructs an optimal hyperplane, utilizing a small set of vectors near a boundary to solve the classification issues (63). Meanwhile, since applying radial basis function (RBF) with the Gaussian functions as the kernel function, SVM can reduce the computational complexity of the training procedure (64). A heuristic “grid search” using 5-fold cross-validation was performed to achieve the best prediction accuracy.

### Model Evaluation

To better evaluate the performance and stability of the models, the original dataset of samples was randomly divided into a calibration set (n_KenyaAA_ = 17, n_Kilimanjaro_ = 20, n _Yunnan−Arabica_ = 24), a validation set (n_KenyaAA_ = 5, n_Kilimanjaro_ = 5, n_Yunnan−Arabica_ = 5), and a prediction set (n_KenyaAA_ = 8, n_Kilimanjaro_ = 5, n_Yunnan−Arabica_ = 7). Although LDA and SVM models conduct 5-fold cross-validation, the same three sets were also used. To evaluate the performance of a model to discriminate specific coffee categories, sensitivity (Sen), specificity (Spe), and accuracy (Acc) of a certain type of coffee were calculated as follows:

(4) $$\begin{array}{r} Sensitivity=\frac{TP}{TP+FN} \end{array}$$

(5) $$\begin{array}{r} Specificity=\frac{TN}{TN+FP} \end{array}$$

(6) $$\begin{array}{r} Accuracy=\frac{TP+TN}{TP+FN+TN+FP} \end{array}$$

where TP is true positive, TN is true negative, FP is false positive, and FN is false negative. Sensitivity represents the ability of the model to correctly identify a specific type of coffee samples, specificity represents the ability of the model to correctly recognize the other two types of coffee samples, and accuracy represents the ability of the model to classify all types of samples correctly.

## Results and Discussion

### Terahertz Spectra Analysis

**Figure 3:**
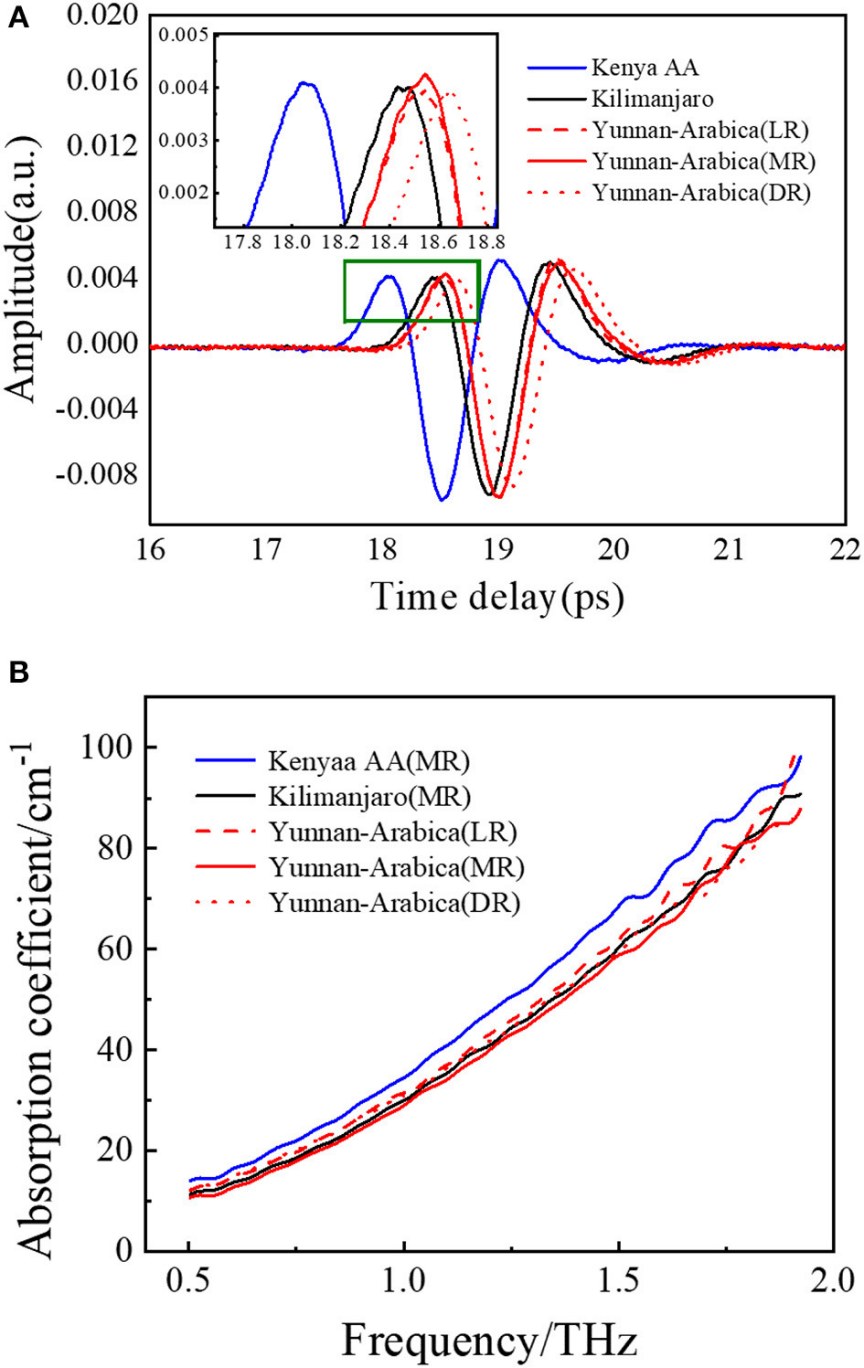
Terahertz (THz) time-domain waveforms **(A)** and THz absorption coefficient spectra **(B)**.

Figure 3A shows the average THz time-domain signals of coffee beans from three different geographical origins. The spectral trends of all samples are similar to the average spectrum, so the average spectrum is used to show the intraspecies consistency and interspecies differences of coffee beans. Although the waveforms are similar, the phase difference between the coffee beans from Kenya and the other two kinds could be observed in the partially enlarged view. The amplitude and the phase of the time-domain signal of Yunan coffee beans with different roasting degrees show a slight difference. In the average absorption spectra (Figure 3B), Kenya coffee beans occupy the largest absorption. There are no obvious absorption peaks within 0.5–1.9 THz, which may due to the fact that, in the complex samples, the molecular interaction with surrounding substances will cause the disappearance of peaks (65). These three kinds of samples show slight differences in the band of 1.5–1.9 THz, which mainly represent the absorption of dry substances, such as hemicellulose, cellulose, fat, lignin, chlorogenic acid, protein, and caffeine. However, it is still difficult to classify the geographical origins directly just by virtue of these minor differences. Therefore, it is necessary to investigate the classification model to help identify the geographical origins of coffee beans.

### Classification Analysis

The redundancy of spectral variables will affect the classification effect of traditional machine learning models, so spectral features were extracted by two data dimensionality reduction methods, including PCA and GA.

**Figure 4:**
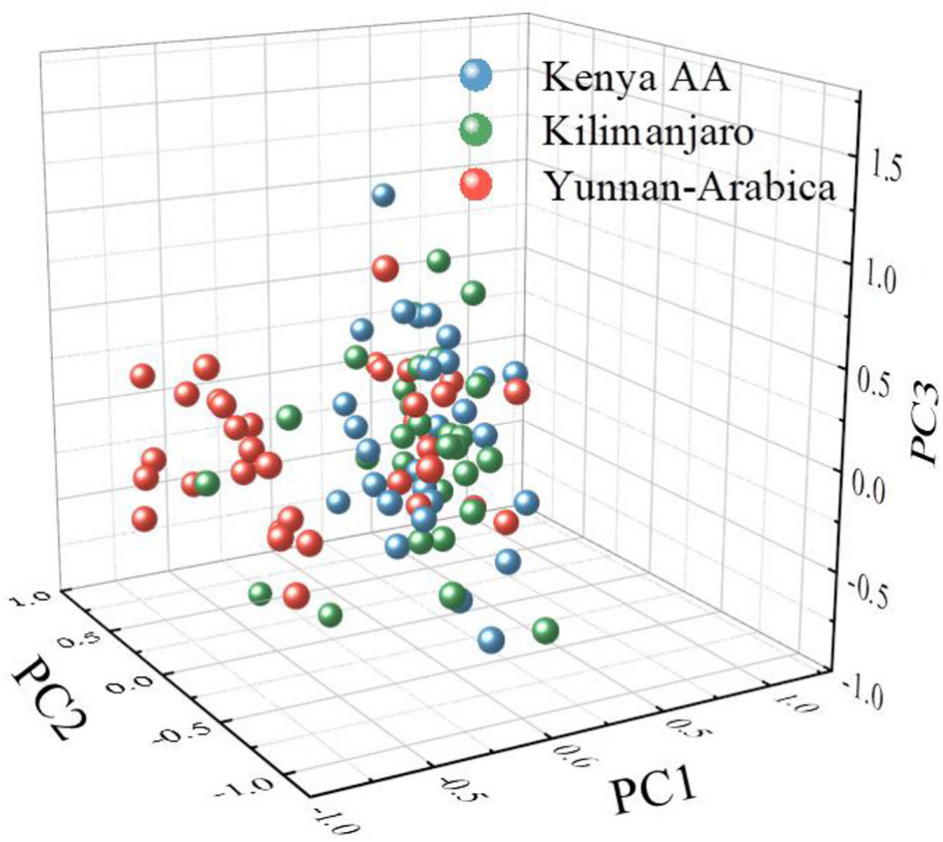
A three-dimensional score plot of the first three principal components (PCs) for the coffee beans from Kenya, Tanzania, and China.

Figure 4 is the PC1, PC2, and PC3 score maps of PCA. As can be seen from the figure, in the PCA model constructed from the overall samples, the total proportion of the first three selected PCs has reached 94.5% (84.4, 5.2, and 4.85%, respectively). However, coffee bean samples from different regions exhibited high overlapping due to the same compositional properties. Therefore, the first three PCs, while characterizing the major part of the THz spectrum differences, are not sufficient to achieve the classification distinction. In this study, the first nine PCs, whose accumulative contribution rate of PCs reached 99.9%, were selected for subsequent analysis.

**Figure 5:**
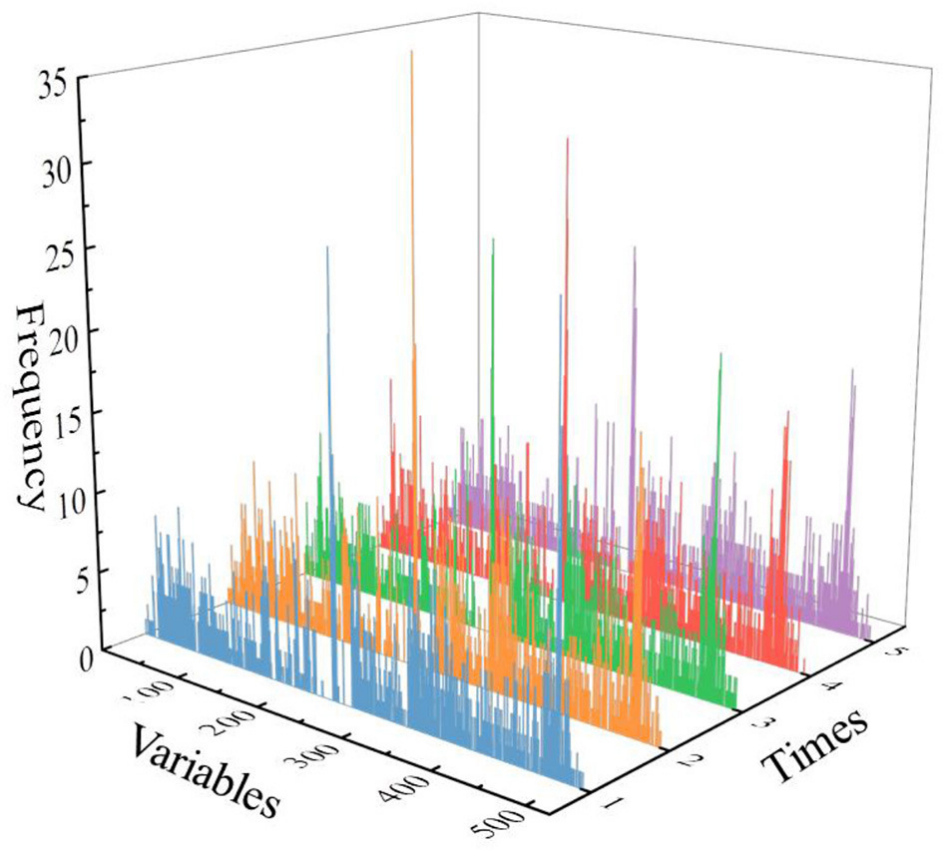
The frequency of every variable of genetic algorithm (GA) for five times.

**Figure 6:**
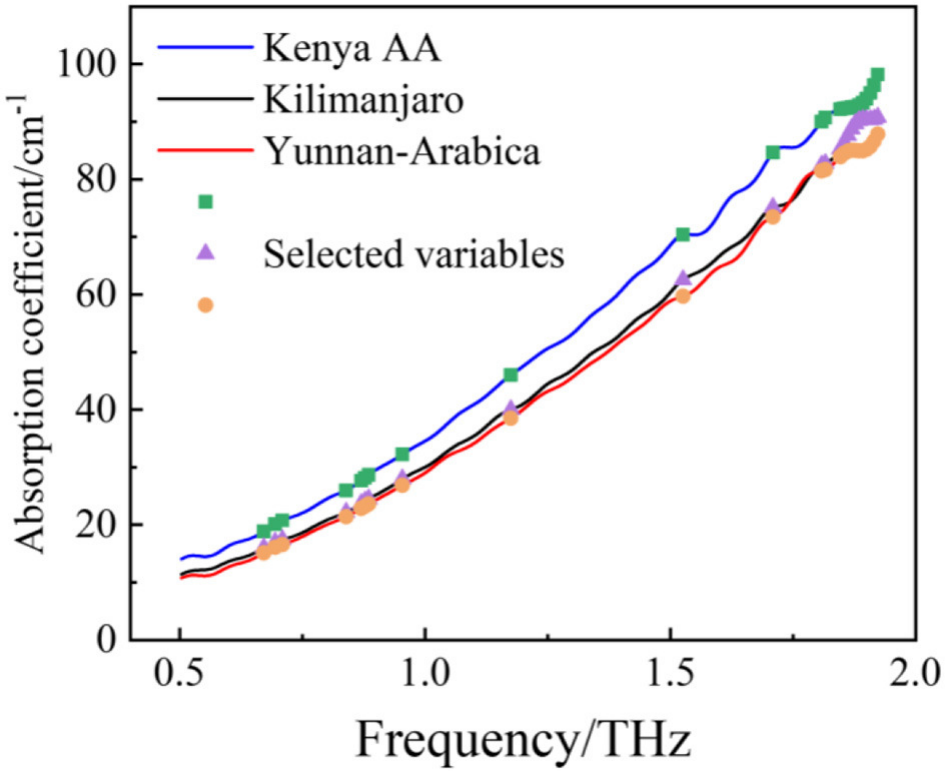
The final result of the GA method for variable selection.

Figure 5 shows the histogram of the frequency of each selected variable. Because of the randomness of the GA, the intersection of five results after running five time was selected as the most streamlined and important variable. Figure 6 shows the final selected variables by the GA method. After the GA process, 21 variables were selected as the most streamlined and important variables. And the majority of the variables selected by the GA method distribute within the ranges of 1.7–1.9 THz, which also corresponds to the frequency band with the largest difference among the three origins of coffee samples in the absorption spectrum.

After the feature extraction process, the spectral variables are imported into traditional machine learning methods. The classification results were compared with the CNN model. Table 2 shows Acc, Sen, and Spe for the calibration, validation, and prediction sets for all classification models that were considered. These metrics were also used to create the confusion matrix of the results as shown in Table 3.

**Table 2:** Comparison of model performance obtained with machine learning methods.

**Classification methods**		**Calibration**			**Validation**			**Prediction**		
	**Feature**	**Sen**	**Spe**	**Acc**	**Sen**	**Spe**	**Acc**	**Sen**	**Spe**	**Acc**
	**extraction**	**(%)**	**(%)**	**(%)**	**(%)**	**(%)**	**(%)**	**(%)**	**(%)**	**(%)**
LDA	None	63.1	82.5	65.6	46.7	73.3	46.7	19.2	58.3	20.0
	PCA	73.1	87.0	72.1	26.7	63.3	26.7	38.2	69.2	40.0
	GA	74.3	87.7	75.4	33.3	66.7	33.3	42.4	71.4	45.0
SVM	None	86.4	92.9	85.2	73.3	86.7	73.3	47.1	75.4	50.0
	PCA	86.4	92.9	85.2	80.0	90.0	80.0	66.5	83.4	65.0
	GA	85.8	93.1	86.9	80.0	90.0	80.0	77.4	88.9	75.0
CNN		95.3	97.6	95.1	93.3	96.7	93.3	90.5	95.0	90.0

**Table 3:** Confusion matrix detailing the multiclass discrimination results of three different geographical origins of coffee beans using genetic algorithm (GA)-support vector machine (SVM) and convolutional neural network (CNN).

**Actual class**	**Predicted class**					
	**GA-SVM**			**CNN**		
	**Kenya**	**Tanzania**	**Yunnan**	**Kenya**	**Tanzania**	**Yunnan**
Kenya	6	2	0	8	0	0
Tanzania	0	5	0	0	5	0
Yunnan	0	3	4	1	1	5
Sen (%)	75.0	100.0	57.1	100.0	100.0	71.4
Spe (%)	100.0	66.7	100.0	91.7	93.3	100.0
Acc (%)	75.0			90.0		

The best classification results were obtained using the CNN model, which reaches 90.0% Acc, 90.5% Sen, and 95% Spe in the prediction set. The most noteworthy result is that the CNN approach outperforms the classifiers built on LDA and SVM. LDA is a linear algorithm that is most capable of processing simple datasets, while SVM is a non-linear approach that specializes in high-variety datasets but depends on the input features. Otherwise, the performance of LDA and SVM could be improved by using the feature extraction method. As shown in Table 2, in LDA and SVM models, the Acc, Sen, and Spe values of the prediction set increase while using the extracted feature compared with the use of the whole spectra. The feature data provide useful information and reduce the chances of overfitting. However, the drawback is that the feature extraction method found to be optimal for one classification model is not guaranteed to work well with other models. There is a one-to-one match between each classification model and the best feature extraction method. For application, it is necessary to test different combinations of feature extraction methods and classification methods to achieve the best classification results.

Nevertheless, this drawback does not occur with a deep learning model, which can analyze different kinds of data. Moreover, in this study, the CNN model demonstrated a powerful classification ability even in using the raw spectral data, which means that deep learning has the potential to be a simple one-step process in classification analysis.

Table 3 depicts the confusion matrix and descriptive statistics related to the classification model. In traditional machine learning models, GA-SVM gets the best accuracy of 75%. The classification results of the CNN model were 90% accurate with a specificity of 100% for Yunnan coffee beans and a sensitivity of 100% for Kenya and Tanzania coffee beans. The coffee beans from Kenya and Tanzania are all correctly distinguished. However, no matter what complex and specific method is adopted to achieve the origins classification, there is still a non-negligible degree of uncertainty. The fact that there has been a strong confusion about the classification of the Yunnan coffee bean samples is unsurprising, as the wrong determination is mostly attributed to the interference of different roasting degrees. In more detail, it may be due to the fact that Yunnan coffee beans were further prepared with three different roasting degrees; Kenya AA and Kilimanjaro coffee beans were both prepared with only one roasting degree. Therefore, the difference between Yunnan coffee beans increases, which makes more difficult for the model to extract accurate features, and then makes the ability of classification model to distinguish Yunnan coffee beans poor.

Results are encouraging because they indicate that the use of THz and deep learning has positive effects and could be the object of application. Additionally, the CNN approach is less sensitive to data preprocessing than SVM and LDA. Nevertheless, the small samples remind us that the optimum CNN classifier has not been achieved yet. Larger samples will be needed for CNN model training to make it more accurate and robust in the future.

## Conclusions

The geographical origin is one of the most relevant factors that determine the quality and commercial value of coffee beans. In this study, popular machine learning algorithms were used to classify the geographical origins of coffee beans, based on THz spectroscopy. A diversity of classification models was evaluated, including CNN, PCA-LDA, GA-LDA, PCA-SVM, and GA-SVM. Among them, above 90% accuracy is reached by using the CNN model. The main advantage of the CNN approach is that there is no need to predefine the feature of THz spectra. Although the neural network takes a long time to train, the well-trained model is available to achieve rapid detection, thus reducing the pretraining time.

In summary, the effective and satisfactory approach to classifying the geographical origin of coffee beans, which taps the potential application of deep learning in the authenticity of agricultural products, expands the application of THz spectroscopy. Future research directions include (1) using a larger database to improve the training process of the network, which includes samples with wider geographic distribution, more diversified varieties, and more roasting conditions; (2) using a more detailed study of the choice of CNN architectures and parameters to find ideal networks for the problem; and (3) introducing transfer learning to make the model suitable for other classification tasks.

## Data Availability Statement

The original contributions presented in the study are included in the article/supplementary material, further inquiries can be directed to the corresponding author/s.

## Author Contributions

SY: conceptualization, methodology, software, and writing—original draft. CL: conceptualization, methodology, formal analysis, writing—review and editing, and funding acquisition. YM: methodology, software, and visualization. WL: methodology and software. RL: validation, formal analysis, resources, and funding acquisition. WC: validation, resources, and funding acquisition. DH: conceptualization, investigation, and resources. KX: formal analysis, resources, funding acquisition, and project administration. All authors contributed to the article and approved the submitted version.

## Conflict of Interest

The authors declare that the research was conducted in the absence of any commercial or financial relationships that could be construed as a potential conflict of interest.
